# Isocitrate dehydrogenase 1 (IDH1) mutation-specific microRNA signature predicts favorable prognosis in glioblastoma patients with IDH1 wild type

**DOI:** 10.1186/1756-9966-32-59

**Published:** 2013-08-29

**Authors:** Zheng Wang, Zhaoshi Bao, Wei Yan, Gan You, Yinyan Wang, Xuejun Li, Wei Zhang

**Affiliations:** 1 Department of Neurosurgery, Beijing Tiantan Hospital, Capital Medical University, No. 6 Tiantan Xili, Dongcheng District, Beijing 100050, China; 2 Beijing Neurosurgical Institute, No. 6 Tiantan Xili, Dongcheng District, Beijing 100050, China; 3 Department of Neurosurgery, Xiangya Hospital, Central South University, Changsha 410008, China

**Keywords:** IDH1, Wild type, MiRNA signature, Glioblastoma

## Abstract

**Background:**

To date, no prognostic microRNAs (miRNAs) for isocitrate dehydrogenase 1 (IDH1) wild-type glioblastoma multiformes (GBM) have been reported. The aim of the present study was to identify a miRNA signature of prognostic value for IDH1 wild-type GBM patients using miRNA expression dataset from the The Cancer Genome Atlas (TCGA).

**Methods:**

Differential expression profiling analysis of miRNAs was performed on samples from 187 GBM patients, comprising 17 mutant-type IDH1 and 170 wild-type IDH1 samples.

**Results:**

A 23-micoRNA signature which was specific to the IDH1 mutation was revealed. Survival data was available for 140 of the GBM patients with wild-type IDH1. Using these data, the samples were characterized as high-risk or low-risk group according to the ranked protective scores for each of the 23 miRNAs in the 23-miRNA signature. Then, the 23 IDH1 mutation-specific miRNAs were classified as risky group and protective group miRNAs based on the significance analysis of microarrays d-score (SAM d-value) (positive value or negative value). The risky group miRNAs were found to be expressed more in the high-risk samples while the protective group miRNAs were expressed more in the low-risk samples. Patients with high protective scores had longer survival times than those with low protective scores.

**Conclusion:**

These findings show that IDH1 mutation-specific miRNA signature is a marker for favorable prognosis in primary GBM patients with the IDH1 wild type.

## Background

MicroRNAs (miRNAs) are short noncoding ribonucleic acid (RNA) molecules, approximately 22-nucleotide long, and single-stranded [1]. MiRNAs are post-transcriptional regulators that bind to complementary sequences on target messenger RNA transcripts (mRNAs), usually resulting in translational repression or target degradation and gene silencing, thereby modulating a variety of biological process such as cell growth, proliferation, differentiation, metabolism, and apoptosis [2-4]. Some miRNAs are reported to be associated with clinical outcomes in some tumors, such as blood carcinomas [5,6], lung cancer [7,8], pancreatic cancer [9,10], and colon adenocarcinoma [11,12].

Glioblastoma (GBM, WHO grade IV glioma) is the most malignant brain tumor in adults. Even after treatment with surgical resection and radiotherapy plus concomitant chemotherapy, most patients with the diagnosis of GBM seldom survive more than 15 months [13]. A number of molecular markers for GBM associated with diagnosis, prognosis, and treatment have been identified. Somatic mutations in IDH1 have been identified in GBM patients, especially in secondary GBM which evolves from lower-grade gliomas [14]. Several miRNA signatures associated with IDH1 mutations have been revealed via miRNA expression profiling and better outcomes have been predicted for GBM patients with IDH1 mutations [1]. However, to date, no valuable prognostic miRNA signatures have been reported for patients with wild-type IDH1 GBM. In the present study, we used the GBM miRNA dataset from The Cancer Genome Atlas (TCGA, http://cancergenome.nih.gov/) and selected miRNAs that were differentially expressed between wild-type and mutant-type IDH1 GBM samples. As a result, we successfully identified a 23-miRNA signature, which predicted a better outcome for GBM patients with wild-type IDH1.

## Methods and materials

### Samples

MiRNA expression data (level 3) and the corresponding survival data for glioblastoma samples were downloaded from The Cancer Genome Atlas (TCGA) data portal. Two mutant-type IDH1 samples and 30 wild-type IDH1 samples were removed during analysis because of unavailable survival information or very short survival time (less than 30 days, probably caused by other lethal factors). Thus, a total of 155 GBM patients, with 15 mutant-type and 140 wild-type IDH1 patients, were enrolled for further analysis. Because the data were obtained from TCGA, further approval by an ethics committee was not required.

Whole-genome microRNA profiles of glioblastoma patient were downloaded from public the Cancer Genome Atlas (TCGA) database (http://cancergenome.nih.gov/).

### Data analysis

Differential expression profiling analysis was performed on the GBM miRNA dataset of TCGA using significance analysis of microarrays (SAM), performed using BRB-ArrayTools developed by Dr. Richard Simon and the BRB-ArrayTools Development Team (available at http://linus.nci.nih.gov/BRB-ArrayTools.html). The differential expression standard was set to 1.5 fold (SAM-d value score greater than 1.5 or less than −1.5) and P-values less than 0.01 were taken as significant. The SAM application calculates a score for each miRNA on the basis of the change of expression relative to the standard deviation of all measurements. To assess the survival prediction value of selected miRNAs, a protective-score formula for predicting survival was developed based on a linear combination of the miRNA expression level multiplied by the SAM d-value. MiRNAs from 155 GBM patients, including 15 mutant-type and 140 wild-type IDH1 samples, that showed enormous differences in expression between the wild-type and mutant-type IDH1 GBM samples, were selected for further analysis.

## Results

### Identification of the 23-miRNA signature

**Figure 1:**
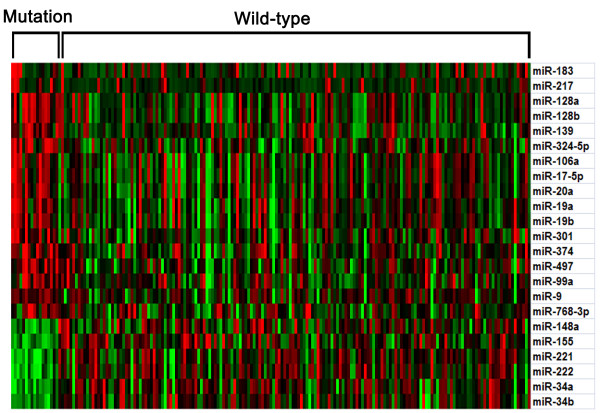
**The IDH1 mutation-specific 23-miRNA signature.** The 23 miRNAs were differentially expressed by more than 1.5 fold in GBM samples with mutant-type IDH1 compared to those with wild-type IDH1.

Twenty-three miRNAs were identified from the total of 470 GBM miRNAs in TCGA and defined as IDH1 mutation-specific miRNA signatures (Figure 1). Each of the 23 miRNAs showed significantly aberrant expression in the mutant-type IDH1 samples and, thus, were defined as a 23-miRNA signature specific to IDH1 mutation.

### Accessing protective scores

**Figure 2:**
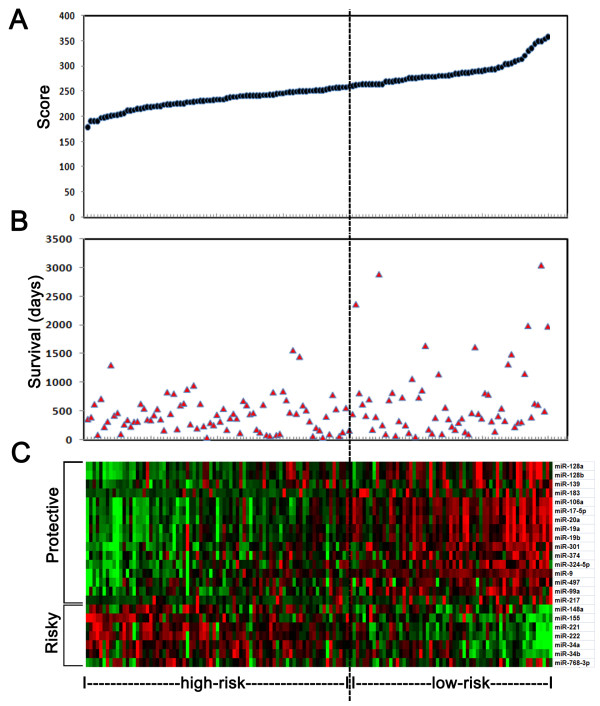
**Protective scores for the 23-miRNA signature and survival days in GBM patients with wild-type IDH1. A.** Ranked protective scores. **B.** Survival days for the 140 GBM patients. **C.** The risky group and protective group for the 23 miRNAs. Risky miRNAs were expressed more in the high-risk group and protective miRNAs were expressed more in the low-risk group.

To assess the value of survival prediction for the 23-miRNA signature protective-scores were calculated for all enrolled GBM patients. The 140 patients with wild-type IDH1 were ranked according to the protective score values for the 23-miRNA signature along with the corresponding survival data (Figure 2B and 2C). Using the 60th percentile protective-score as a cutoff, the 140 wild-type IDH1 samples were divided into two groups, high-risk (corresponding to the low-score group) and low-risk group (corresponding to the high-score group) (Figure 2A and 2C).

The 23 miRNAs were divided into two groups according to the SAM d-value (positive value or negative value), the risky group and the protective group with 16 and seven miRNAs, respectively (Figure 2C). Protective miRNAs were expressed at higher levels in the low-risk group, while risky miRNAs tended to be expressed more in the high-risk group (Figure 2C).

**Figure 3:**
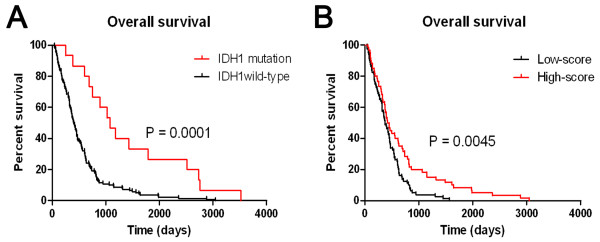
**Overall survival of GBM patients in the mutant-type and wild-type IDH1 groups. A.** Patients with mutant-type IDH1 had much better outcome than those with wild-type IDH1. **B.** Kaplan-Meier curves for the low-score and high-score groups. In the 140 IDH1 wild-type GBM patients, patients in the high-score group had much longer overall survival times than those in the low-score group.

We also compared the overall survival of the patients in the mutant-type (15 samples) and the wild-type IDH1 groups (140 samples) and found statistically significant differences between them (Figure 3A, P = 0.0001). Kaplan-Meier curves for the low-score and high-score groups were shown in Figure 3B. A statistically significant difference was observed between the two groups (P = 0.0045). Patients in the high-score group had better outcomes than patients in the low-score group. Thus, the 23-miRNA signature, which was specific to IDH1 mutation in the GBM samples, may be a marker of favorable prognosis in wild-type IDH1 GBM patients.

## Discussion

Primary GBM is considered to be the most lethal brain tumor in adults. The prognosis is variable, with some patients surviving less than a year and others surviving for three years or more [13]. To date, only IDH1 mutation and O-6-methylguanine-DNA methyltransferase (MGMT) promoter methylation have been identified as stable prognostic indicators for GBM patients across various studies. IDH1 mutations were reported to have a strong positive correlation with overall survival in secondary and primary GBMs, although the mutation rate in primary GBM was much lower than that in secondary GBM [14]. Through differential miRNA expression profiling, we identified a 23-miRNA signature that was implicated with outcomes for GBM patients with the mutant-type IDH1. Nevertheless, until now, no miRNA signature that could serve as an indicator for GBM in patients with IDH1 wild-type is available.

Here, we used a scoring method to measure the relative expression levels of the 23 miRNAs. Then we divided all of the samples into high-score and low-score groups as shown in Figure 2. We found that the high-score group had better clinical outcomes than the low-score group. According to the SAM-d value, these miRNAs were defined as risky miRNA group and protective miRNA group. Seven miRNAs were designated as risky miRNAs, of which higher expressions indicated worse outcomes, and 16 miRNAs were designated protective miRNAs, of which higher expressions indicated better outcomes for GBM patients.

A recent study, which examined the expression data of 305 miRNAs from 222 GBM samples in TCGA dataset, identified a 10-miRNA prognostic signature [15]. The 10-miRNA signature is partially consistent with the 23-miRNA signature that we identified in the present study. The two signatures share six miRNAs, including are protective miRNAs (miR-20a, miR-106a, miR-17-5p) and three risky miRNAs (miR-221, miR-222, miR-148a). To some extent, the overlap between the two miRNA signatures verified the potentially clinically predictive significance of, at least, the 6-miRNA signature. A possible explanation for why the two signatures did not agree exactly may be because of differences in the target population and/or the entry criteria. In another study, a 5-miRNA signature was identified as a prognostic biomarker in Chinese patients with primary GBM [1]. This 5-miRNA signature (miR-181d, miR-518b, miR-524-5p, miR-566, and miR-1227) was significantly associated with improved overall survival for GBM patients. Interestingly, none of the five miRNAs in this signature overlapped with the miRNAs in our 23-miRNA signature, probably because different patient populations and datasets were used in the two studies.

We further investigated the six miRNAs that were common to the 10-miRNA and 23-miRNA signatures. Some studies have shown that miR-183 was significantly down-regulated in osteosarcoma and may subsequently promote migration, invasion, and recurrence of osteosarcoma [16]. In our study, we found that miR-183 was a favorable predictor for GBM, which was consistent with its effect in osteosarcoma. In advanced colorectal cancer, miR-148a expression was the most significantly downregulated, which resulted in a worse therapeutic response and poor overall survival [17]. A similar effect was found in GBM, and, in our study, miR-148a was classified as one of the risky biomarkers for GBM. In a study of adult T-cell leukemia, miR-155 was identified as a novel unfavorable biomarker for disease progression and prognosis [18]. Another study reported that elevation of plasma miR-155 was associated with shorter survival times in non-small cell lung cancer [19]. These findings were consistent with our results for the function of miR-155. MiR-221 and its paralogue miR-222 are known inhibitors of angiogenesis, which act by blocking cell migration and proliferation in endothelial cells [20,21]. Other studies have reported different functions for miR-221, suggesting that miR-221 was also associated with induction of angiogenesis [22,23]. In our research, miR-221 and miR-222 were identified as unfavorable indictors for GBM. In a study into chronic lymphocytic leukemia, miR-34a and miR-17-5p were found to be downregulated in chronic lymphocytic leukemia patients with tumor protein p53 (TP53) abnormalities, indicating that higher expression levels of miR-34a and miR-17-5p may predict a better clinical outcome for these patients [24].

In TCGA, the IDH1 mutation-type samples account for only 10–16% of the GBMs, most of which are secondary GBMs. Our results provided a robust clinical prognostic indicator for GBM patients with wild-type IDH1. However, we still have no idea how exactly this 23-miRNA signature worked in GBM. Clearly, the mechanisms behind the roles of these miRNAs require further investigation. Better insights into how the 23-miRNA signature functions in GBM will potentially contribute to an understanding of the genetic aberrations that are involved in tumor genesis, progression, and/or response to treatment. In particular, there are a number of significant advantages over microarray methodologies for the routine examination of miRNA signatures. Analysis can be undertaken straightforwardly, rapidly and cost-effectively. It is much more applicable and feasible to be tested in the clinical practice than whole genome miRNA profiling. Furthermore, these profoundly aberrantly expressed miRNAs can serve as potential molecular targets for new therapeutic strategies, subsequently leading to improved outcomes for GBM patients.

## Competing interest

The authors declared that have no competing interest.

## Authors’ contributions

ZB and ZW collected the dataset and drafted the manuscript together. WY and GY performed the data analysis work and help with making the figures. YW made the figures. XL and WZ conceived the study and revised the manuscript. All authors read and approved the final manuscript.
